# A Theoretical Framework for Evaluating Psychiatric Research Strategies

**DOI:** 10.1162/CPSY_a_00008

**Published:** 2017-12-01

**Authors:** Kentaro Katahira, Yuichi Yamashita

**Affiliations:** 1 Department of Psychology, Graduate School of Informatics, Nagoya University, Nagoya, Aichi, Japan; 2 Department of Functional Brain Research, National Institute of Neuroscience, National Center of Neurology and Psychiatry, Tokyo, Japan

**Keywords:** psychiatric research, statistical power, generative model, research domain criteria, diagnostic category

## Abstract

One of the major goals of basic studies in psychiatry is to find etiological mechanisms or biomarkers of mental disorders. A standard research strategy to pursue this goal is to compare observations of potential factors from patients with those from healthy controls. Classifications of individuals into patient and control groups are generally based on a diagnostic system, such as the *Diagnostic and Statistical Manual of Mental Disorders (DSM)* or the *International Classification of Diseases* (*ICD*). Several flaws in these conventional diagnostic-based approaches have been recognized. The flaws are primarily due to the complexity in the relation between the pathogenetic factors (causes) and disorders: The current diagnostic categories may not reflect the underlying etiological mechanisms. To overcome this difficulty, the National Institute of Mental Health initiated a novel research strategy called Research Domain Criteria (RDoC), which encourages studies to focus on the neurobiological mechanisms and core aspects of behavior rather than to rely on traditional diagnostic categories. However, how RDoC can improve research in psychiatry remains a matter of debate. In this article, we propose a theoretical framework for evaluating psychiatric research strategies, including the conventional diagnostic category-based approaches and the RDoC approach. The proposed framework is based on the statistical modeling of the processes of how the disorder arises from pathogenetic factors. This framework provides the statistical power to quantify how likely relevant pathogenetic factors are to be detected under various research strategies. On the basis of the proposed framework, we can discuss which approach performs better in different types of situations. We present several theoretical and numerical results that highlight the advantages and disadvantages of the strategies. We also demonstrate how a computational model is incorporated into the proposed framework as a generative model of behavioral observations. This demonstration highlights how the computational models contribute to designing psychiatric studies.

## INTRODUCTION

One of the major challenges of basic research in psychiatry is to find etiological mechanisms or biomarkers that can be helpful for investigating the treatment or predicting the prognosis of a mental disorder. A standard research strategy for achieving this goal is as follows. Researchers first classify the individuals into a clinical population (patient group) and a nonclinical population (control group). This classification is generally based on the current diagnostic systems, such as the *Diagnostic and Statistical Manual of Mental Disorders* (*DSM*; American Psychiatric Association, 2013) or the *International Classification of Diseases* (*ICD*; World Health Organization, 1993). Then, researchers attempt to determine the candidate pathogenetic factors (e.g., genetic variants, neural activity, or environmental and social milieu) that significantly differ between groups. The current diagnostic systems are based on multiple criteria of symptoms or signs. For example, for an individual to be classified into major depressive disorder (MDD), he or she should have several (at least five) symptoms, such as depressed mood, anhedonia, fatigue, change in appetite, feelings of worthlessness, and suicidal thoughts.

Several methodological flaws have been recognized in the conventional category-based approaches (e.g.,Cuthbert & Kozak, 2013; Insel et al., 2010; Owen, 2014. One notable flaw is the heterogeneity in the population assigned with the same diagnostic label. The heterogeneity of the corresponding biological and social factors in a population may reduce the likelihood of detecting such factors. This heterogeneity can arise from two sources. The first is the heterogeneity of symptoms in a population with the same diagnostic labels. For example, patients diagnosed with schizophrenia can have only positive symptoms or also have negative symptoms. The second source of the heterogeneity is diversity in the cause of similar symptoms, namely, multiple etiological causes can lead to similar observable outcomes, a property termed *equifinality* (Flagel et al., 2016). Another flaw is that similar symptoms that may share similar pathogenetic factors are included in different categories of mental disorders, which may lead to comorbidity between disorders with different diagnostic labels. In addition, a single common genetic risk factor can be associated with multiple mental disorders (e.g., Cross-Disorder Group of the Psychiatric Genomics Consortium, 2013). This property is termed *multifinality*; that is, a single cause can have divergent outcomes (Flagel et al., 2016). These problems can be summarized as the lack of a strict one-to-one mapping from pathogenetic factors to the current category of the mental disorders, and there appear to be many-to-one or one-to-many mappings between them. These issues can obscure the understanding of the etiological causes of mental disorders.

To overcome the aforementioned problems, the U.S. National Institute of Mental Health (NIMH) established the Research Domain Criteria (RDoC; Cuthbert, 2014; Insel, 2014; Insel et al., 2010). RDoC recommend that researchers seek the relationships among the behavioral measurements (included as “behavior” and “self-reports” in the unit of analysis) and biological and social factors (included as “genes,” “molecules,” “cells,” “circuits,” and “physiology” in the unit of analysis), focusing on a specific research domain and construct. The research domains (e.g., “positive valence systems”) contain constructs (e.g., “reward learning”). The RDoC approach is not supposed to use the conventional categories of the diagnostic systems. The analysis method is dimensional rather than categorical, which means that the RDoC approach deals with behavioral observations (including those regarded as “symptoms” in a traditional view) with continuous measures without categorical boundaries between healthy individ uals and patients. In the RDoC approach, independent variables might be specified from any level of the unit of analysis, with dependent variables chosen from one or more other levels of analysis. If we assume linear relationships between measures in the units of analysis, a typical statistical approach is regression or correlation analysis. The relationship, however, is not necessarily linear and can be nonlinear (e.g., inverted U-shaped curve; Cuthbert, 2014). Although we only focus on linear correlations in this article, our framework can be extended to a nonlinear case.

In addition, the RDoC approach does not treat symptoms as a syndrome (a group of signs and symptoms that occur together) as the category-based approach does. The RDoC approach encourages the investigation to focus on minimum behavioral elements and underlying mechanisms. In this regard, the spirit of RDoC includes that of cognitive neuropsychiatry, which is the field that attempts to understand symptoms in mental disorders as aberrations of cognitive functions (David & Halligan, 2000; Halligan & David, 2001).

However, how RDoC can improve research in psychiatry remains a matter of debate. Although there are methodological flaws in the current diagnostic systems (*DSM*/*ICD*), as discussed above, the *DSM*/*ICD* systems also provide advantages. One advantage is that the reliability of the diagnosis can be increased by using multiple criteria. This may lead to an increase in the likelihood that a researcher finds the pathogenetic factors of the mental disorders, in contrast to the RDoC approach, which decomposes symptoms into distinct dimensions. Therefore it is important to clarify under which conditions the RDoC approach outperforms the conventional category-based approaches. For this purpose, mathematical and computational models may provide a useful framework for quantitatively addressing such questions.

In this study, we propose a theoretical framework for examining how effective research strategies, including those encouraged by RDoC, are in basic research in psychiatry. The proposed framework evaluates how effectively each method finds a target pathogenetic factor given some situational settings. In this framework, we first construct a formal, hypothetical generative model of symptoms and their causes. From the generative model, synthesized samples are generated. Then, we evaluate how likely several research methodologies are to detect the true cause of a symptom or pathological behavior from observed data based on standard statistical methods, including *t* test, correlation analyses, and regression analyses. We particularly focus on the statistical power, which is the probability that the statistical hypothesis test detects the effect of the pathogenetic factor if the effect actually exists. Note that the proposed framework is not intended to be used as a data analysis tool; rather, the framework predicts how a research strategy performs given a hypothetical generating process of a mental disorder. In addition, note that the evaluation of the research strategy based on the proposed framework depends on a generative model, which represents the hypothesis about the mechanisms of disorders. We discuss this issue later.

One issue that our framework addresses is whether a mental disorder is best investigated using categorical or dimensional approaches (First, 2016). The conventional diagnostic approach (with *DSM* or *ICD*) is by definition categorical: It determines whether an in dividual has a disorder using diagnostic criteria. Although diagnostic categories are useful for communication between clinicians, between researchers, and between a clinician and a patient, dimensional approaches have been recognized to have advantages. One advantage that is closely related to the present work regards statistical power. It has been reported that categorical approaches in which the originally continuous data are converted into categorical variables by splitting the sample at some point degrade the statistical power (Altman & Royston, 2006, Cohen, 1983; MacCallum, Zhang, Preacher, & Rucker, 2002). This issue has also been discussed in the context of psychiatry (Kamphuis & Noordhof, 2009; Kraemer, 2007; Kraemer, Noda, & O’Hara, 2004). Our proposed framework is related to these previous works, but it has an important difference. Our method focuses on the way in which the subjects are sampled, not only on how the given samples are analyzed. We assume that the category-based approach samples the subjects such that both the patient group and the control group have sufficient numbers of subjects with predefined diagnostic criteria. In contrast, the dimensional approach is assumed to sample subjects randomly from a population. Thus the samples to be analyzed are different between these two approaches. We discuss how the difference in the sampling methods affects the statistical power.

The remainder of this article is organized as follows. First, we formally explain the proposed framework. Next, we provide some simulation results that highlight the properties of the proposed framework. Finally, we discuss the implications of the results and the limitations of the proposed framework.

## Proposed framework

Here we formally describe the proposed framework. We assume that there are *N* potential pathogenetic factors that can be the causes or predictors of behavioral observations (including symptoms). The *j*th pathogenetic factor is denoted as *x*_*j*_. All the pathogenetic factors are summarized as a column vector: ***X*** = [*x*_1_, … , *x*_*N*_]^*T*^, where ⋅^*T*^ denotes the transpose. Here the pathogenetic factors may include specific alleles or brain connectivity, which can be predictors of risk. The factors may also include the dysregulation of a neuromodulator or neurotransmitter, which can be a target of medical treatment, as well as environmental and social miliex. The measurement of the value of variable *X* is often contaminated by noise that may be caused by estimation error or measurement error. The measured or estimated value of *x*_*j*_ is denoted as ${\hat{x}}_{j}$. In reality, the pathogenetic factors themselves have interactions and constitute a causal network. However, the first step of basic research may identify components of the network simply by comparing each factor between the patient group and the control group. The following cases intend to model this initial phase of psychiatric research.

We consider *M* behavioral observations, denoted as ***Y*** = [*y*_1_, … , *y*_*M*_]^*T*^. Here the behavioral observations include self-reported symptoms and signs that are used in *DSM*/*ICD*-based classifications and the scores of some behavioral tests. In RDoC, such behavioral observations are included in the units of analysis “behavior” or “self-reports.”

### Mapping From Pathogenetic Factors to Behavioral Observations

The next step is to define the generative model, which represents how the pathogenetic factors are translated to behavioral observations. We assume that the pathogenetic factors ***X*** are translated to behavioral observations ***Y*** via some function *f* with some added noise *ϵ*. In vector form, this can be written as

Y=f(X)+ϵ, (1)

where ***ϵ*** is an *M*-dimensional column vector and *f*(⋅) represents a map from an *N*-dimensional column vector to an *M*-dimensional column vector. The noise may include the individual difference in resilience, any other personality trait that affects how easily the individual experiences the disorder, or the errors in the subjective report and behavioral observation.

In the following simulations and analyses (Cases 1–5), we consider a simple model with linear mapping and Gaussian noise, called the linear Gaussian model. The mathematical formulation of this model is presented in Katahira and Yamashita (2017, Appendix A). In the linear Gaussian model, each pathogenetic factor is assumed to be independently drawn from a Gaussian (normal) distribution. Each behavioral observation *y*_*i*_ is the linear combination of ***X*** and the Gaussian noise ***ϵ***_*i*_. From this assumption, each *y*_*i*_ also obeys the Gaussian distribution, which means that behavioral observations are continuous variables. Many inclusion criteria in the current diagnostic systems (i.e., *DSM* and *ICD*) take on discrete values (e.g., existence or absence of a symptom). For such cases, *y*_*i*_ may be interpreted as a behavioral phenotype, based on which a psychiatrist or a patient makes decisions regarding each symptom rather than the symptom itself. In Case 6, rather than a linear Gaussian model, a reinforcement learning model is used as a generative model for behavioral observations related to psychosis.

### Category-Based Approach

**Figure 1.**
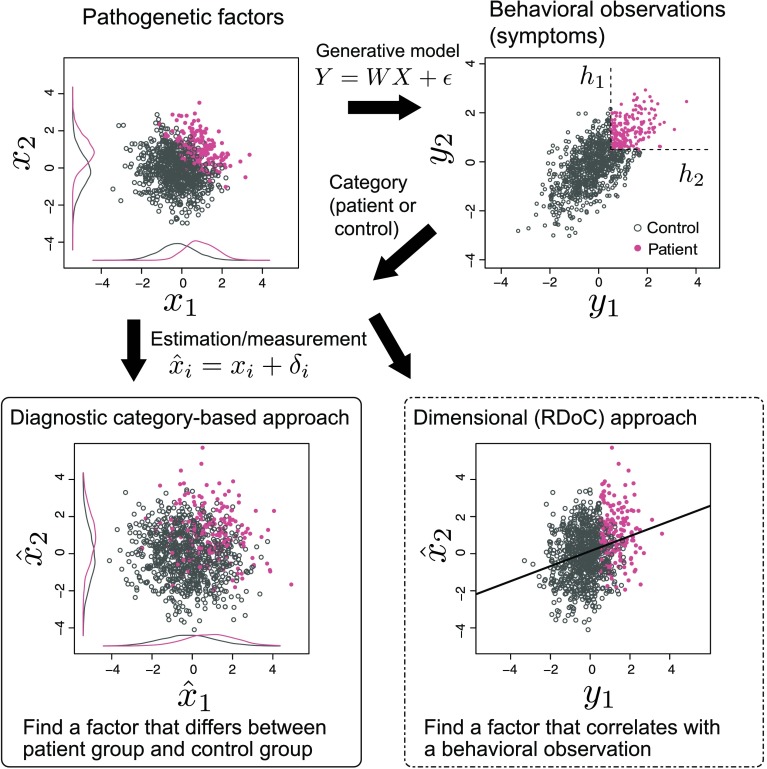
**Schematic of the proposed framework.** Each dot represents an individual. Here the samples were generated by the linear Gaussian model. First, the value of pathogenetic factors *x*_1_ and *x*_2_ are generated from a Gaussian distribution independently. Then, these factors are transformed into behavioral observations *y*_1_ and *y*_2_ with linear mapping ***Y*** = *W* ***X*** and adding some noise *ϵ*. The individuals are classified as patients if both behavioral observations *y*_1_ and *y*_2_ have larger values than *h*_1_ and *h*_2_, respectively (here we used the common cutoff point *h*_1_ = *h*_2_ = 0.5). The red dots represent the individuals of the patient group, and the gray dots represent the individuals of the control group. The conditional distributions of *x*_1_ and *x*_2_ given groups are plotted in the top left panel (gray lines for the control group and red lines for the patient group). Note that the conditional distributions no longer obey the Gaussian distribution. These pathogenetic factors are assumed to be observed with adding some estimation error *δ*, which also obeys a Gaussian distribution. The diagnostic category-based approach attempts to find a pathogenetic factor *x*_*j*_ whose observed value ${\hat{x}}_{j}$ differs between the patient group and control group (bottom left panel). The dimensional approach attempts to find a factor *x*_*j*_ that correlates with a behavioral measure *y*_*i*_ without using a category label (bottom right panel).

In the proposed framework, the category-based approach first classifies the individuals into the patient group or the control group, depending on the values of their behavioral observation ***Y***. For example, if *y*_*i*_ for all *i* exceeds the cutoff point *h*_*i*_ (*y*_*i*_ ≥ *h*_*i*_ ∀_*i*_), the individual is classified into the patient group (in Figure 1, the individuals indicated with red dots belong to the patient group). Except for Case 2, where we examine the effect of the margin between the patient group and control group, individuals who have any behavioral observation that does not satisfy the criterion (*y*_*i*_ < *h*_*i*_ ∃_*i*_) are classified into the control group (the individuals indicated with gray dots in Figure 1). Note that a subject is classified into a patient group only when all of the *M* behavioral observations exceed the threshold. In this article, we do not consider a polythetic criteria approach, in which the diagnosis is assigned when a patient exhibits a designated number from a list of symptoms provided for each disorder category (e.g., five of nine for MDD). Such a polythetic criteria approach can be incorporated in the proposed framework. For simplicity, however, we leave them as a future work. In the following simulations, we set *h*_*i*_ = *h* = 0.5 ∀_*i*_ unless otherwise stated.

The category-based approach seeks the pathogenetic factors that significantly differ between the two groups. The estimated or measured value of *x*_*j*_, which is denoted by ${\hat{x}}_{j}$, is assumed to contain a measurement error. This error is modeled by adding a Gaussian variable *δ*_*j*_ to the true value *x*_*j*_ (see Katahira & Yamashita, 2017, Appendix A). Note that we formally and explicitly model the measurement or estimation error rather than incorporate a specific estimation process.

In the simulation, the samples of subjects (*n*_1_ subjects from the control group and *n*_2_ subjects from the patient group, resulting in a total of *n*_1_ + *n*_2_ = *n* subjects) are randomly selected from both groups, and their observed pathogenetic factors $\hat{x}$ are subjected to an unpaired *t* test with the equal variance assumption. The null hypothesis is that the population means of ${\hat{x}}_{j}$ for the patient group and that for the control group are the same. If the significance of the difference is detected at the significance level *α* (*α* = 0.01 for Cases 1-5 and *α* = 0.05 for Case 6), then the factor *x*_*j*_ is considered to be a pathogenetic factor relevant to the mental disorder. When multiple candidate factors are submitted to statistical tests, a correction should be made for multiple comparisons (e.g., Bonferroni correction) to suppress family-wise error rates. However, for simplicity, we do not perform such a correction in this article. Incorporating a correction is straightforward and does not influence the quantitative results reported herein.

When more than one pathogenetic factor can be observed at the same time, one may use logistic regression, in which the objective variable is diagnostic category (0 control, 1 patient) and the regressors (predictors) are the observed values of potential pathogenetic factors $\hat{x}$. For this case, the hypothesis test can be performed for each pathogenetic factor with the null hypothesis for which the regression coefficient for the factor is zero. In all the cases that we considered, however, we found that logistic regression provided a lower statistical power compared with an independent *t* test while showing a similar tendency with a *t* test. Thus we do not present the logistic regression results.

### Dimensional (RDoC) Approach

The dimensional approach addresses the behavioral observations, including symptoms and pathogenetic factors, without categorical labels. This approach utilizes the natural variation of the population. RDoC basically encourages the dimensional approach. In the proposed framework, the dimensional approach is simulated by sampling *n* subjects from the population irrespective of the behavioral phenotype (symptom). A statistical hypothesis test is then conducted. When there is one measured potential pathogenetic factor, one simple approach tests the null hypothesis that the correlation coefficient between *y*_*i*_ and ${\hat{x}}_{j}$ is zero. When the correlation is significant (the null hypothesis is rejected), then the *j*th pathogenetic factor *x*_*j*_ is deemed to be a factor that is relevant to the behavioral observation *y*_*i*_.

When more than one potential pathogenetic factor can be observed at the same time, one may use multiple linear regression, in which the objective variable is each behavioral measure *y*_*j*_ and the regressors are potential pathogenetic factors $\hat{x}$. For this case, the hypothesis test can be performed for each pathogenetic factor with the null hypothesis that the regression coefficient for the factor is zero. We will report the multiple linear regression results in Case 1 and Case 5. In reality, all of the true causes of the generative process of mental disorders that may be included in the generative model are rarely observed in a single study: Researchers can observe only part of them. Thus, in general, the statistical model in the analysis of the observed data would be simpler than the generative model: the number of pathogenetic factors that can be included in the analysis is less than the number of those included in the generative model. Case 5 also considers this situation.

RDoC is more than just a dimensional approach to mental disorders. It emphasizes the consideration of constructs of the mental disorders based on neurobiological grounding. In addition, RDoC encourages investigations that target multiple units of analysis. The targeted units of analysis can all be biological ones (e.g., “cells” and “circuits”). However, to relate the biological mechanisms to mental disorders, it at least requires knowledge that relates some biological factors to some behavioral observations as a starting point. Additionally, RDoC is intended to create a novel discrete category of mental disorder after sufficient research progress. Thus the dimensional approach in the present framework models are intended to capture only one aspect of the RDoC approach, particularly at the beginning phase.

## Results

Below, we provide the results of simulations based on our framework. The first simple cases (Cases 1-1 and 1-2) demonstrate what types of suggestions the proposed framework can provide. The next four cases (Cases 2–5) highlight the basic theoretical properties of the proposed models. Readers not interested in the theoretical details may wish to skip these four cases. The model settings in Cases 1–5 are intended to illustrate the common structures of basic research in psychiatry rather than to model a specific disorder. In the final case (Case 6), we present a concrete example of considering a specific disorder (i.e., psychosis) by incorporating a computational model into the proposed framework.

In general, the statistical power monotonically increases as the sample size (the number of subjects) increases (e.g., see Figure 4C). The order of statistical powers of the methods that we consider in this article does not depend on the sample size. However, the extreme value of sample size makes the differences smaller. Thus we selected the total sample size as *n* = 40 (except for Case 2) such that the differences among methods manifest.

### Case 1: Multiple Disorder Categories

**Figure 2.**
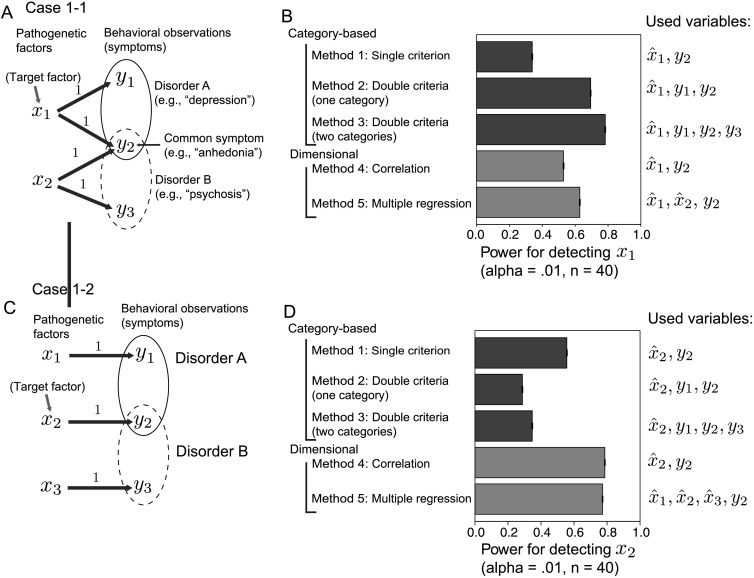
**Case 1-1 and Case 1-2: Two representative cases with two disorder categories.** A) Schematic of the generative model of Case 1-1. This case assumes that there are two distinct pathogenetic factors corresponding to each disorder category. The common symptom *y*_2_ has different pathogenetic factors *x*_1_ and *x*_2_. B) The statistical power of several approaches for detecting the target factor *x*_1_ (Case 1-1). Method 1, Method 2, and Method 3 employ category-based approaches, which sample individuals based on some criteria and perform the statistical test of the difference in the means (*t* test). Method 1 uses the single behavioral observation *y*_2_. This samples 20 individuals with *y*_2_ < *h* for the control group and 20 individuals with *y*_2_ ≥ *h* for the patient group. Method 2 uses the double criteria. The subjects in the patient group satisfy *y*_1_ ≥ *h* and *y*_2_ ≥ *h*. The subjects in the control group do not satisfy either of these criteria. Method 3 uses both disorder criteria, and individuals who fall into Disorder A but not Disorder B are sampled for the patient group. The control group has neither Disorder A nor Disorder B. Method 4 and Method 5 are dimensional approaches that sample the individuals randomly and perform a correlation analysis (Method 4) or a multiple regression (Method 5). The error bars indicating the 95% confidence intervals of the power estimate are plotted, although they are almost invisible because their length is very short. C) Schematic of the generative model of Case 1-2. This case assumes that there are three distinct pathogenetic factors, where each affects a single symptom. The common symptom *y*_2_ has a single pathogenetic factor *x*_2_. D) The statistical powers of several approaches for detecting the target factor *x*_2_ (Case 1-2). The convention is identical with Case 1-1.

The first two cases (Cases 1-1 and 1-2) consider the basic problem in psychiatric research. Consider that there are two disorder categories, which we call Disorder A and Disorder B (e.g., depression and psychosis, respectively). For simplicity, we assume that each disorder has two symptoms and that one of these symptoms is shared by both disorders (Figures 2A and 2C). Behavioral observation *y*_2_ represents the common symptom between the two disorders (e.g., anhedonia), and *y*_1_ and *y*_3_ are specific symptoms for each disorder. Assume that the goal of the researcher is to detect the pathogenetic factor of the common symptom *y*_2_. An individual is assumed to be diagnosed with Disorder A if *y*_1_ ≥ *h* and *y*_2_ ≥ *h*; here *h* is the common cutoff point, and we set *h* = 0.5. He or she is also diagnosed with Disorder B if *y*_2_ ≥ *h* and *y*_3_ ≥ *h*. Comorbidity (a single individual can be diagnosed with both Disorder A and Disorder B) is allowed. We calculated the statistical powers of five methods (Methods 1–5) for detecting the target factor. The category-based approaches (Methods 1–3) compare the observed pathogenetic factor (${\hat{x}}_{j}$) between the control group and the patient group. Methods 1–3 differed in terms of the inclusion criteria for the two groups. We also consider the category-based analysis using only a single criterion, which compares individuals with *y*_2_ ≥ *h* and those with *y*_2_ < *h* (Method 1).

Here we consider two possible etiological mechanisms for this situation. Case 1-1 assumes that there are two distinct pathogenetic factors for each disorder category (Figure 2A). In this case, the common symptom arises from different pathogenetic factors, denoted as *x*_1_ and *x*_2_. Figure 2B shows the statistical powers of several analyses for detecting the target factor *x*_1_. In this case, the category-based analysis using only a single criterion (Method 1) did not yield a good result: Half of the trials failed to obtain the significant difference in ${\hat{x}}_{1}$ (at the critical level, *α* = 0.01; top bar of Figure 2B).^1^ In contrast, the category-based approaches using two symptoms as criteria (Methods 2 and 3) provided a better result. In Method 2, the patient group consists of individuals who satisfy the criteria of Disorder A, whereas the control group consists of the other individuals. Method 2 yielded a significant difference with a probability of greater than 60% (Figure 2B).

**Figure 3.**
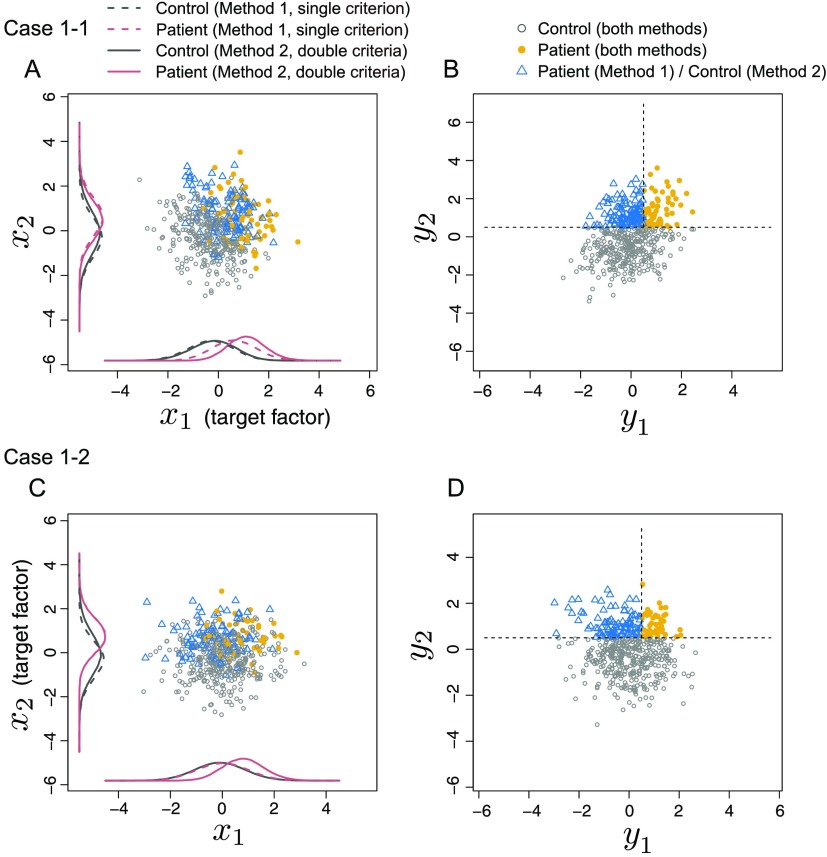
**Sample scatterplots that explain how category-based approaches perform differently in Case 1-1 (A–B) and Case 1-2 (C–D).** The orange-filled circles represent the individuals classified as patient by both Method 1, which uses a single symptom, and Method 2, which uses double symptoms. The blue triangles represent the individuals classified as patient only by Method 1 but not by Method 2. Thus these individuals belong to the control group in Method 2. The individuals marked with gray dots belong to the control group in both methods. The marginal distributions of *x*_1_ and *x*_2_ for each group for each method (dashed line: Method 1; solid line: Method 2) are drawn in (A) and (C).

The reason for the difference between the statistical powers of Method 1 and Method 2 can be understood by checking the distributions of samples for each group in each method (Figure 3A). Method 2 includes only individuals with *y*_1_ ≥ *h* and *y*_2_ ≥ *h*, which are indicated with orange-filled circles in Figures 3A and 3B. In contrast, Method 1 also includes the individuals with *y*_2_ ≥ 1 but *y*_1_ < *h*, who are marked with blue triangles. As shown in Figure 3A, the individuals marked with blue triangles tend to have smaller values of *x*_1_ compared to the individuals marked with orange filled circles. Thus, Method 2, which employs only the individuals indicated with orange filled circles, improves the discriminability compared to Method 1, which includes the individuals marked with blue triangles (dashed lines in Figure 3A). The discriminability is measured using Cohen’s *d*, which is defined as the difference between two means divided by the pooled standard deviation (see Katahira & Yamashita, 2017, Appendix B). Method 1 yielded *d* = 1.06 for *x*_1_ (*d* = 0.71 for ${\hat{x}}_{1}$), whereas Method 2 yielded *d* = 1.61 for *x*_1_ (*d* = 1.02 for ${\hat{x}}_{1}$), which is a remarkable improvement in discriminability. This result is directly related to the statistical power: The larger *d* is, the greater the power is (note that an actual statistical test is performed for the measured/estimated value ${\hat{x}}_{1}$ rather than for the true value *x*_1_).

Method 3 is a diagnostic category-based approach that utilizes the information about both Disorder A and Disorder B: Individuals in the patient group are with Disorder A but without Disorder B, whereas individuals in the control group are without both disorders. Method 3 provided slightly higher power than Method 2 (Figure 2B). This result is because the patients with Disorder A but without Disorder B have more specificity of pathogenetic factor *x*_1_ compared to those with both disorders. If the individuals have Disorder B, then they tend to have a higher value of *x*_2_. Having a larger common symptom value *y*_2_ may be due to this higher value of *x*_2_ and not due to *x*_1_. Excluding such individuals and choosing patients only with Disorder A helps to illuminate the effect of *x*_1_.

Dimensional approaches, which test the correlation between ${\hat{x}}_{1}$ and *y*_2_ (Method 4), yielded greater power than Method 1 but less power than the two category-based approaches (Methods 2 and 3). Including both pathogenetic factors in the explanatory variables of multiple regression increased the power (Method 5), but it did not exceed the power of the two category-based approaches.

Case 1-2, which assumes a one-to-one mapping from the pathogenetic factors to the symptoms, produced quantitatively different results. In this case, there is one distinct pathogenetic factor for each symptom. The use of the disorder categories with multiple criteria did not increase the power. In contrast, the analyses using double criteria (Methods 2 and 3) yielded lower power compared to the category-based analysis using only a single criterion (*y*_2_ ≥ *h*; Method 1). The reason for the deterioration in the power of the double criteria methods can also be understood by examining the distributions of samples (Figure 3C). Method 2 includes the individuals with *y*_2_ ≥ *h* but *y*_1_ < *h*, who are marked with blue triangles, in the control group, whereas Method 1 classified these individuals into the patient group. In this case, the individuals indicated with blue triangles also have higher values of *x*_2_, which are comparable to those of individuals indicated with orange dots. Thus including the subjects marked with blue triangles pulls the distribution of *x*_2_ of the control group (black solid line in Figure 3B) toward that of the patient group (red solid line), which reduces the discriminability. Method 1 yielded *d* = 1.40 for *x*_2_ (*d* = 0.89 for ${\hat{x}}_{2}$), whereas Method 2 yielded *d* = 0.99 for *x*_2_ (*d* = 0.66 for ${\hat{x}}_{2}$). Moreover, in this case, dimensional approaches, which test the correlation between ${\hat{x}}_{2}$ and *y*_2_ (Method 4), yielded greater power than the category-based approaches. Here including other variables in the multiple regression did not increase the power (Method 5).

The implications of the results are as follows. The diagnostic categories based on the syndrome can be useful for detecting the pathogenetic factor if the factor is related to those symptoms (as in Case 1-1). However, if there is no such one-to-many mapping, using multiple criteria is not useful: Using a single observation is more efficient for detecting the underlying factor (as in Case 1-2). Among the methods based on a single behavioral observation, the dimensional (correlation) approach provides greater statistical power (compare Method 1 and Method 4 in Figures 2B and 2D). These properties are systematically investigated in the following analyses (Cases 2 and 3).

### Case 2: Category-Based Versus Dimensional Approaches

**Figure 4.**
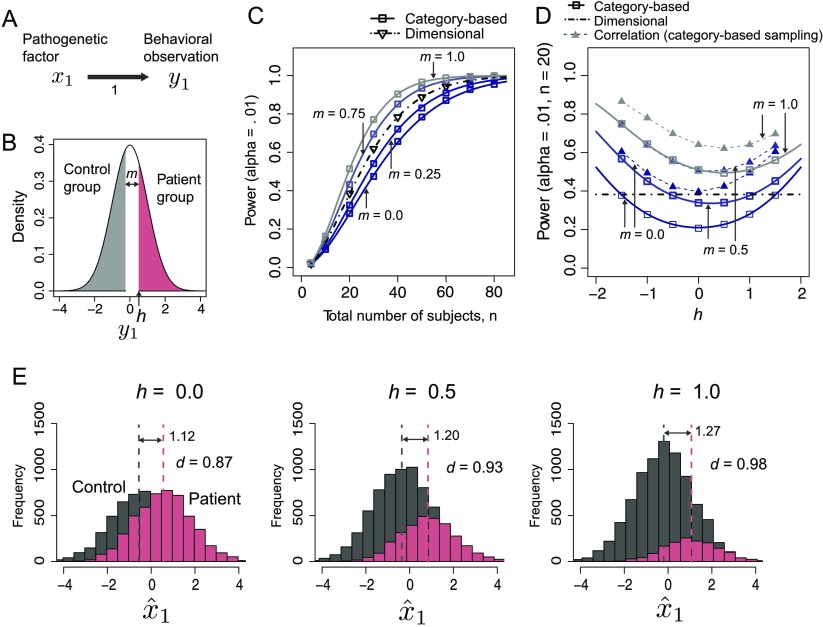
**Comparison of the statistical powers of the category-based and dimensional approaches in Case 2.** A) The schematic of the generative model in Case 2. This case includes a single pathogenetic factor (*N* = 1) and a single behavioral observation (*M* = 1). B) Illustration of the category-based approach with a margin. C) The statistical power (with the significance level α = 0.01) of both methods as a function of the total number of subjects, with a variable margin *m* for the category-based approach. The lines represent the results obtained from analytical calculations (see Katahira & Yamashita, 2017, Appendix B). The symbols represent the results of the Monte Carlo simulations (see Appendix A). D) The effect of cutoff point *h*. The results of the standard dimensional approach, which does not use the cutoff point, are indicated by the horizontal chain line. The results of the dimensional (correlation) approach using the sample obtained from the category-based method (given *h*) are plotted by the dashed lines with inverted triangles. E) The distribution of the estimated pathogenetic factor ${\hat{x}}_{1}$ for three *h* cases (with *m* = 0). The sample means of each group are indicated by the vertical dashed lines. *d* = Cohen’s *d*.

In Case 2, we compared the statistical powers of the category-based approach and the dimensional (correlation) approach for the simplest case in which there is a single pathogenetic factor (*M* = 1) and a single behavioral observation (*N* = 1). This case is intended to examine the result of Case 1, which shows that the dimensional (correlation) approach provides higher statistical power when one behavioral observation is used. The model structure is illustrated in Figure 4A. Here we also examined the effect of the margin (denoted as *m*) between the patient group and control group. Suppose that the individuals with *y* less than *h* − *m* are classified into the control group and that the individuals with *y* larger than *h* are classified into the patient group (Figure 4B). The individuals with *y* falling into the margin are not included in the study. Actual samples in psychiatry studies may include such a margin: Researchers may exclude individuals who are not classified into the clinical group but present behavioral phenotypes that are close to the cutoff point.

Figure 4C shows the power with which the pathogenetic factor *x*_1_ is detected as a function of the total number of individuals. For this case, the statistical powers of both methods can be analytically obtained (see Katahira & Yamashita, 2017, Appendix B; Figure 4C, solid lines for the category-based approach and chain line for the dimensional approach). The symbols (squares for the category-based approach and triangles for the dimensional approach) represent the numerical results obtained from 100,000 runs of the Monte Carlo simulations. The analytical results (lines) perfectly agree with those obtained from the simulations (symbols), thereby validating the analytical calculations. The results indicate that if there is no margin (*m* = 0), then the dimensional approach (using correlation coefficients) yields a higher power compared to the category-based approach (using the unpaired *t* test). This result is because the correlation coefficients can utilize full information on the magnitude of *x*_1_, whereas the category-based approach ignores the information of the distribution within the group. If there is a margin, then the category-based approach can outperform the dimensional approach. With a larger margin, the category-based approach can distinguish clusters in the distribution *x*_1_ while suppressing the impact of the noise added to *x*_1_. However, note that with a larger margin, it becomes more difficult to find samples for the control group.

In the preceding results, the cutoff point was fixed to *h* = 0.5. Next, we varied the cutoff point *h* to examine how it affects the power. The results are shown in Figure 4D. As *h* moved from zero, the power increased. The model yields minimum power when the distribution of the control group and patient group is symmetric (which occurs when *h* − *m*/2 = 0). Figure 4E shows an example of the histogram of ${\hat{x}}_{1}$. As the cutoff point *h* became large, the difference between the means of the two groups increased (from 1.12 to 1.27). Consequently, as *h* moved away from the mean of the population, the effect size (Cohen’s *d*) and statistical power increased.

Note that the samples to be analyzed are different between the two approaches. The dimensional approaches randomly draw samples from the population without any inclusion criteria. In Case 2, if the same sample (which is sampled by the category-based approach) was used for both of the approaches, then the dimensional (correlation) approach always provided superior power to the category-based approaches (Figure 4D, dashed lines with filled triangles).

### Case 3: The Effect of the Number of Diagnostic Criteria in the Category-Based Approach

**Figure 5.**
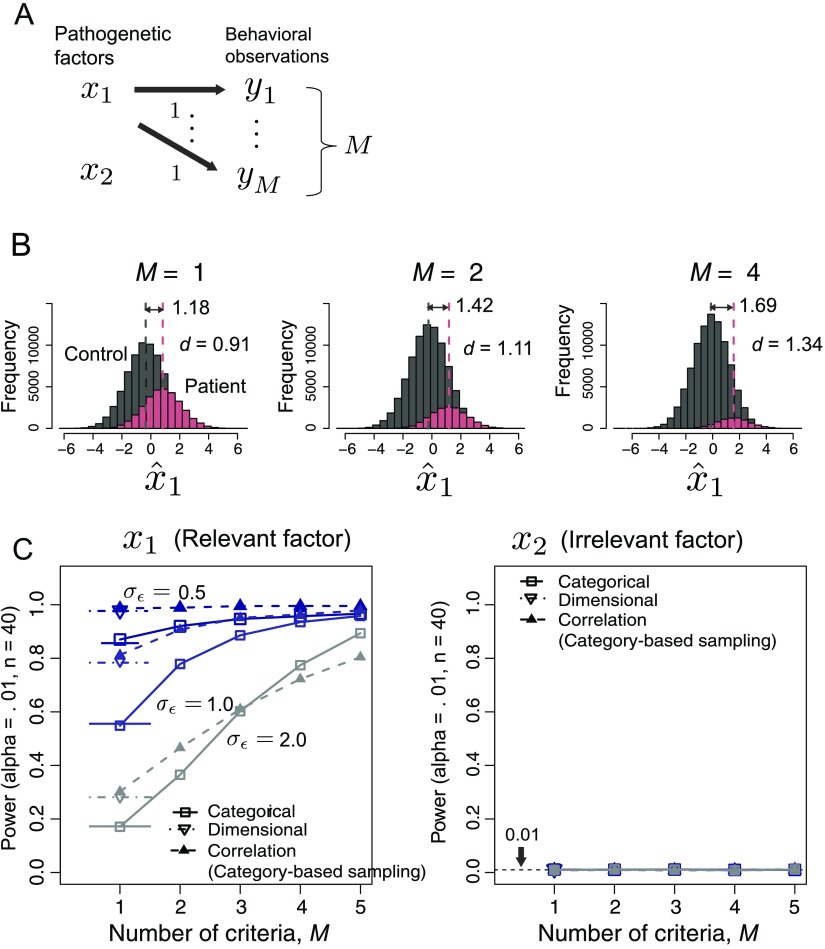
**The effect of the number of diagnostic criteria *M* in the category-based approach (Case 3).** A) The schematic of the generative model in Case 3. Here the model includes two pathogenetic factors (*N* = 2; *x*_2_ is irrelevant) and *M* behavioral observations. B) The distribution of the estimated pathogenetic factor ${\hat{x}}_{1}$ for three *M* cases, with *σ*_*ϵ*_ = 1.0. The sample means of each group are indicated by the vertical dashed lines. *d* = Cohen’s *d*. C) The statistical power (with significance level α = 0.01) of both methods as a function of *M*, with varying standard deviation of the noise σ_ϵ_. The horizontal lines at *M* = 1 represent the analytical results (see Katahira & Yamashita, 2017, Appendix B). The symbols and the lines connecting the symbols for *M* for the category-based approach represent the results of Monte Carlo simulations. Dashed lines with triangles show the power of the dimensional (correlation) approach that used the same sample as the corresponding category-based method.

As shown in Case 1-1, if more than one symptom has a common pathogenetic factor, then including such symptoms in a single disorder category for the category-based approach improves the power for detecting the factor compared to the approach based on a single symptom. In Case 3, we systematically examined the effect of using multiple criteria that share a common pathogenetic factor. Figure 5A shows the structure of the generative model. We assumed that there are two pathogenetic factors (*N* = 2): *x*_1_ is a factor relevant to the mental disorder and is of interest, and *x*_2_ is irrelevant to the mental disorder (Figure 5A). We included the irrelevant factor to examine the false-positive rate, which is the probability that the analysis mistakenly identifies the irrelevant factor as significant. The weight of *x*_1_ for behavioral observation *y*_*j*_(*j* = 1, … , *M*) is set to 1 and that of *x*_2_ is set to zero.

The standard deviation of the noise *σ*_*ϵ*_ and the number of behavioral observations *M* were varied in the simulations. Recall that for a subject to be classified into a patient group, all of the *M* observations need to exceed the threshold. Examples of the histograms of ${\hat{x}}_{1}$ are presented in Figure 5B. The larger the number of criteria *M* is, the greater the difference in the means of the two groups is. Consequently, the larger *M* is, the greater the discriminability and thus the statistical powers are (Figure 5B and Figure 5, left). The power can exceed that of the dimensional approach in which a single behavioral observation is used. These effects are especially prominent when the noise level is high (*σ*_*ϵ*_ = 2.0).

Here the samples submitted to analysis are again different between the category-based approach and the dimensional approach. If the same category-based samples are submitted in both of the approaches, then the dimensional (correlation) approach provides higher power than the category-based approach, particularly when *M* is small (Figure 5C, filled triangles). However, as *M* increases, the power of the category-based approach can even exceed the power of the dimensional approach. The reason for this result is as follows. The power of the correlation approach is influenced by the noise in the chosen behavioral observation *y*_*i*_. The cat egorical analysis uses *y*s only for classifying the individuals. The categorical analyses are also affected by the noise in each *y*_*i*_, but the effect is reduced when the number of *y*s increases.

For the irrelevant factor *x*_2_, the fraction of the factor deemed significant was kept to the preset significance level of 0.01 (Figure 5C, right). This result confirms that the false-positive rate for the irrelevant factor is maintained, as we intended.

### Case 4: The Effect of a Mixture of Pathogenetic Factors

We now discuss the case in which a single behavioral observation *y*_*i*_ is affected by more than one pathogenetic factor *x*_*j*_. It is conceivable that a larger mixture degree leads to difficulty in detecting each pathogenetic factor. For simplicity, we consider the case with two pathogenetic factors and two behavioral observations (*N* = 2 and *M* = 2).

**Figure 6.**
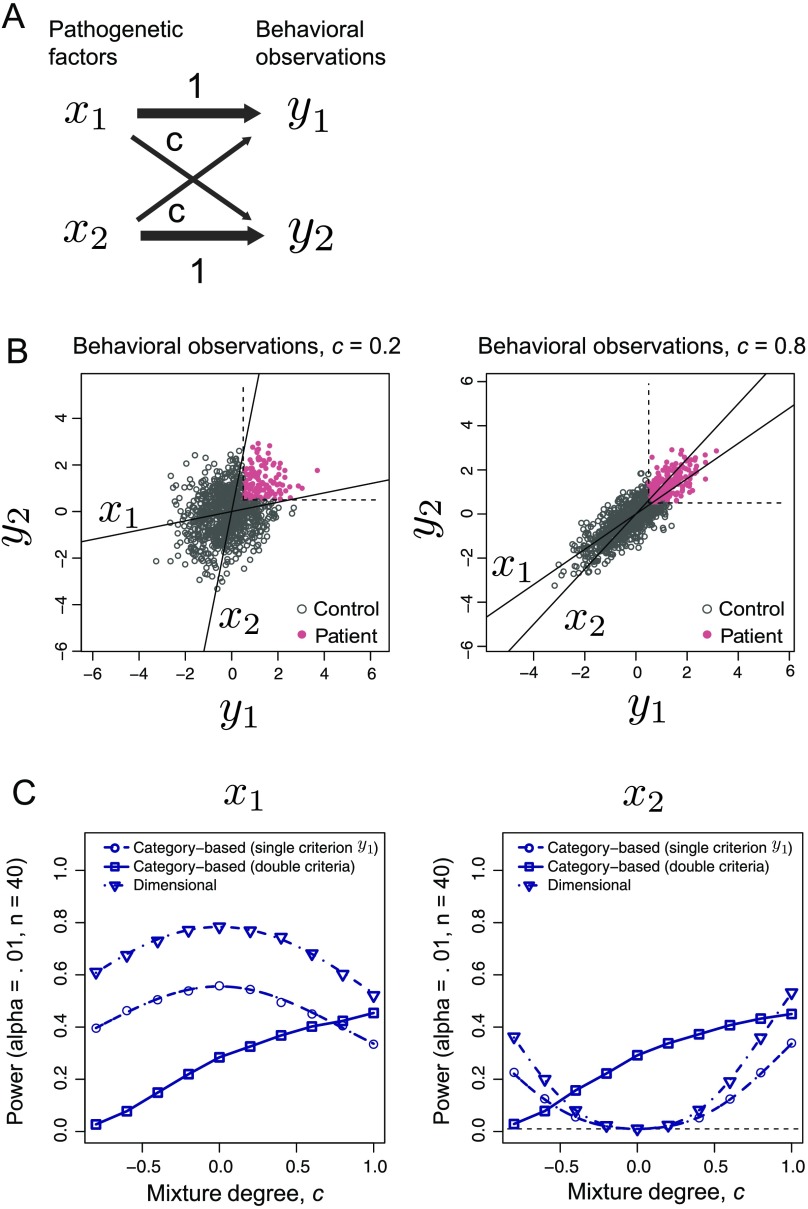
**The effect of a mixture of pathogenetic factors (Case 4).** A) The schematic of the generative model in Case 4. Here the model includes two pathogenetic factors (*N* = 2) and two behavioral observations (*M* = 2). The parameter *c* indicates the mixture degree. B) The scatterplot of *Y* for two *c* cases. C) The statistical power (with critical value α = 0.01) of both methods as a function of the mixture degree *c*. The dash-dotted lines for the dimensional approach and the dashed lines for the category-based approach with a single criterion (*y*_1_ ≥ *h*) denote the results from the analytical calculations (see Katahira & Yamashita, 2017, Appendix B). Symbols and solid lines for the category-based approach using two criteria represent the results of the Monte Carlo simulations.

The transformation matrix is parametrized using a parameter *c* that represents the mixture degree (Figure 6A; also see Eq. (13) in Katahira & Yamashita, 2017, Appendix A). When *c* = 1, *x*_1_ and *x*_2_ equally contribute to both behavioral observations *y*_1_ and *y*_2_ (complete mixture). When *c* = 0, *x*_1_ and *x*_2_ independently contribute to *y*_1_ and *y*_2_, respectively (no mixture). When *c* < 0, *x*_1_ and *x*_2_ have opposite effects on the behavioral measures (one has a positive impact, whereas the other has a negative impact). The effect of *c* on the transformation from pathogenetic factors to behavioral observations is illustrated in Figure 6B.

We consider two cases in the category-based approach: One uses only a single behavioral observation *y*_1_ as a criterion, and the other uses both behavioral observations. The resulting statistical powers are plotted in Figure 6C. As the mixture degree *c* departs from zero in either the positive or negative direction, the power for the methods using a single criterion (*y*_1_ ≥ *h*; dimensional approach and category-based approach) decreases. This result is because the other pathogenetic factor functions as noise in terms of detecting the target *x*_*j*_ when *c* has a nonzero value. In contrast, the power of the category-based approach using two criteria monotonically increases as *c* increases, even when *c* is negative. The reason for this behavior is as follows. This approach equally uses *y*_1_ and *y*_2_; *c* does not largely change the total information extracted from *y*_1_ and *y*_2_. The increase in the power for positive *c* is due to the noise reduction effect reported in Case 3. In contrast, when *c* is negative, individuals whose *x*_1_ is higher can easily be classified into the control group because of the inhibition from other *x*_2_. Distributing subjects with higher values of *x*_1_ into two groups makes the discrimination difficult, thus reducing the statistical power.

The additional pathogenetic factor *x*_2_ is added to *y*_1_ when *c* is nonzero; thus *x*_2_ is detected as a relevant pathogenetic factor even when the single criterion *y*_1_ is used (Figure 6C, right panel).

### Case 5: The Effect of the Number of Pathogenetic Factors

**Figure 7.**
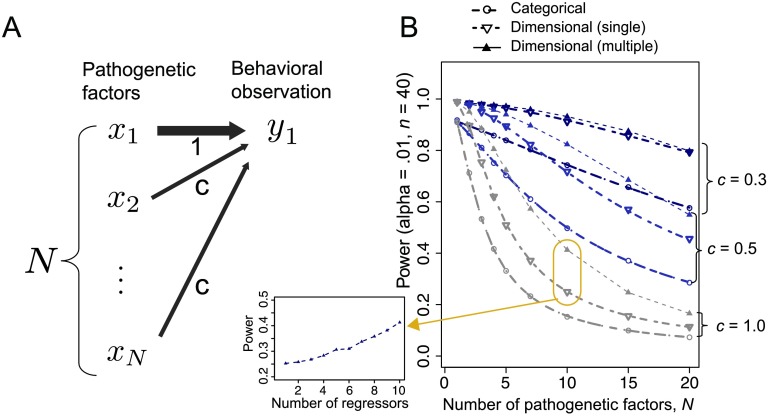
**The effect of the number of pathogenetic factors *N*.** A) The schematic of the generative model in Case 5. The model includes *N* pathogenetic factors and one behavioral observation (*M* = 1). B) The statistical power for detecting the first pathogenetic factor *x*_1_ (with critical value α = .01) as a function of *N*. The dash-dotted lines (for the category-based approach with a single criterion and the dimensional approach with a single regressor) represent the results obtained from analytical calculations. The symbols represent the results of the Monte Carlo simulations. The results of multiple regression using all *N* variables are marked with filled triangles. Only for the case with *N* = 10, the results of the intermediate situation between simple regression and full multiple regression, which uses a part of the observation of 10 factors (including the observed target factor, ${\hat{x}}_{1}$), are shown in the left small panel.

The effect of the mixture reported in Case 4 was not drastic because there were only two pathogenetic factors (*N* = 2). As the next simulation shows, when *N* is large, the effect is large: It is more difficult to detect the individual pathogenetic factor *x*_*j*_. We varied *N* and the mixture degree *c* while keeping the number of behavioral criteria fixed to *M* = 1 (Figure 7A). We consider the statistical power for detecting the first pathogenetic factor *x*_1_, which is assumed to be the target of analysis.

The results are presented in Figure 7B. Overall, the influences of the number of pathogenetic factors *N* and the mixture degree *c* are similar for both the category-based and dimensional approaches. When the mixture degree is maximum (*c* = 1), the statistical power drastically decreases as the number of pathogenetic factors increases. This decrease is modest when the mixture degree is small (e.g., *c* = 0.3). Of course, when there is no mixture (*c* = 0), the statistical power does not depend on the number of pathogenetic factors (data not shown).

Thus far, we have considered the situation in which researchers can measure only a single pathogenetic factor (out of many factors) in one study. Thus we have focused on the simple correlation, or equivalently, simple linear regression analysis. However, large-scale psychiatry studies, such as genome-wide analysis, may measure multiple factors (alleles) in a single study (e.g., Cross-Disorder Group of the Psychiatric Genomics Consortium, 2013). For such situations, multiple regression analysis, which includes more than one candidate pathogenetic factor as regressors, may be used. The filled triangles in Figure 7B show the results of an ideal analysis in which all *N* pathogenetic factors are used as regressors. Here the power for detecting *x*_1_ is computed based on the null hypothesis that the regression coefficient for ${\hat{x}}_{1}$ is zero. These results provided greater statistical power than the simple regression (correlation) analysis did, which used only ${\hat{x}}_{1}$ as a regressor, particularly when *c* was large. This result is because multiple regression can estimate the weight of *x*_1_, suppressing the influence of other confounding factors on the behavioral observation. We also examined the intermediate situation between single regression and full multiple regression: The case in which only *L* (< *N*) potential pathogenetic factors (including the target factor *x*_1_) are available. The power for the case with *N* = 10 is shown in the left small panel of Figure 7B. As expected, the power monotonically increased as the number of regressors increased.

### Case 6: Incorporating a Computational Model

Thus far, we have considered simple linear Gaussian models as models of generative processes of behavioral observations. In reality, the generative processes must be more complex. Using computational models may provide an explicit and more realistic form of the generative processes (Kurth-Nelson et al., 2016). To illustrate how a computational model can be incorporated into our framework, we consider opponent actor-learning (OpAL), a variant of the reinforcement learning model proposed by Collins and Frank (2014). Using this model, Maia and Frank (2017) attempted to explain how the symptoms in schizophrenia emerge from the aberrant activities of dopamine (DA) neurons.

The details of the model are provided in Katahira and Yamashita (2017, Appendix C). In short, this model assumes that several symptoms, including positive and negative symptoms, arise from two types of aberrant activity of DA neurons: (1) increased spontaneous DA transients (parametrized by *p*_SDT_), which can cause DA activity at inappropriate times, and (2) decreased tonic DA level (parametrized by *τ*). Increased spontaneous DA transients induce the aberrant valuation of thoughts, which is assumed to be a cause of delusion. A decreased tonic DA level induces diminished engagement with high-cost activities, which is assumed to correspond to avolition. We synthesized a dataset through a simulation using the model. Specifically, we varied the two parameters that characterize the DA activities and measured behavioral observations. One is a positive symptom, namely, the existence of dominant thought (action), which is caused by aberrant valuation, and the second is a negative symp tom, namely, the diminished engagement with high-cost activities. Using these data, we computed the statistical power for detecting two DA parameters as pathogenetic factors for the symptoms.

**Figure 8.**
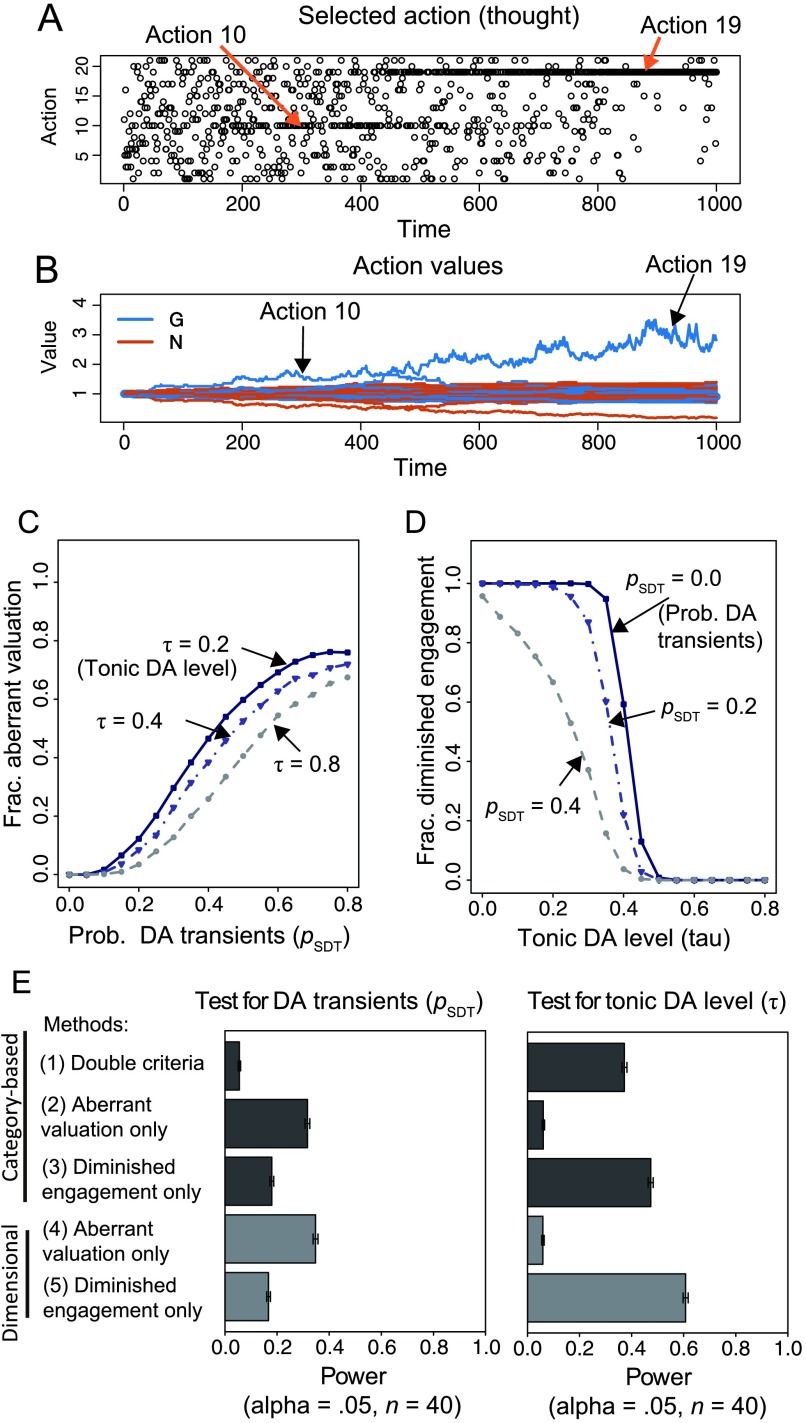
**Demonstration of using a computational model, that is, opponent actor-learning (OpAL), as a generative model of psychosis (Case 6).** A, B) An example episode of the behavior of OpAL as a model of aberrant valuation of thoughts. The DA parameters were set at τ = 0.4 and *p*_*SDT*_ = 0.4. A) Chosen action (thought). Chosen ac tion for each time unit is marked with an open circle. B) Action values. Go values (*G*; blue) and NoGo values (*N*; red) are plotted for all actions. Although the actual reward is not given (*r* = 0), the action values fluctuate due to spontaneous DA transients. C) The effects of the tonic DA level τ and the probability of spontaneous DA transients *p*_*SDT*_ on the fraction of subjects who have the aberrant valuation of thoughts. D) The effects of τ and *p*_*SDT*_ on the fraction of subjects who show diminished engagement with high-cost activities. E) The statistical powers (with the significance level α = 0.05) for detecting the spontaneous DA transients *p*_*SDT*_ (left panel) and the tonic DA level τ (right panel) of several approaches based on different ways of defining “patient” according to symptoms (see Katahira & Yamashita, 2017, Appendix C). The error bars indicate the 95% confidence interval.

Figures 8A and 8B present an example of the formation of dominant thought through the aberrant valuation. Because we set the actual reward to always be zero, any thought (action) should not be reinforced in the standard reinforcement learning. However, the spontaneous DA transients, which occur with probability *p*_SDT_(= 0.4), allow the prediction error signal (denoted by *δ*) to have a nonzero value. Consequently, a randomly chosen thought (action) can be reinforced even without an actual reward. In this simulation, from approximately the 200th time step to approximately the 500th time step, Action 10 was reinforced and frequently selected. After approximately the 400th time step, Action 19 was reinforced and subsequently became dominant. We consider this dominance of a few thoughts without external reinforcement as an occurrence of the aberrant valuation of thoughts (i.e., a positive symptom). An individual is assumed to experience 1,000 discrete time steps. We counted the episode with aberrant valuation of a thought if there at least one thought (action), except for the default action, is selected for 20% of the time steps in the later 500 time steps.

A negative symptom, a diminished engagement with high-cost activities, is examined in the simulation as follows (for details, see Katahira & Yamashita, 2017, Appendix C). An OpAL, as a model of an individual, is given 100 independent opportunities for taking an effortful action. The action values for the effortful action in the model are fixed such that they can represent a high cost of the action by assigning higher values to the NoGo value compared to the Go value. The model can choose not to take that action. Individuals whose choice of taking the effortful action is less than 50% are deemed as having diminished engagement with the high-cost activity (i.e., negative symptom, avolition). The DA level exerts an influence on the probability of taking the effortful action through balancing the weights of the Go value and the NoGo value: A higher DA level imposes more weight on the Go value than on the NoGo value.

Figures 8C and 8D present the relation between the DA parameters and behavioral observations (the fraction of individuals who have each “symptom” out of 1,000 individuals). Overall, the probability of spontaneous DA transients *p*_SDT_ has a direct positive influence on the development of aberrant valuation of thoughts (dominance of a thought), whereas the tonic DA level *τ* has a direct negative relation with how likely the individual is to show diminished engagement with high-cost activities. The tonic DA level *τ* has an inhibitory influence on how likely the individual is to develop a dominant thought (Figure 8C). Meanwhile, the larger *p*_SDT_ is, the less the individuals tend to show diminished engagement with high-cost activities (Figure 8D). These results correspond to the situation in which two pathogenetic factors have opposite effects on each symptom, such as Case 4, where the degree of the mixture *c* is negative. Thus, as we can predict from the results of Case 4, using both criteria in the category-based approach provided a lower power compared to the category-based approaches using each criterion independently (Figure 8E). The results suggest that researchers should treat positive symptoms and negative symptoms separately to find the specific aspect of DA dysfunctions as a pathogenetic factor of the symptoms. In addition, we can hypothesize that the information about the aberrant valuation of thoughts is more effective for detecting spontaneous DA transients as a pathogenetic factor, whereas the measurement of diminished engagement with high-cost activities can be a better variable to be related to the tonic DA level.

The statistical power of the approaches based on the aberrant valuation of thoughts is relatively weak compared to that based on diminished engagement with high-cost activities. This result occurs because the relation between the probability of DA transients *p*_SDT_ and the emergence of dominant thought are highly stochastic: Even when *p*_SDT_ is very large, more than 20% of individuals do not show an aberrant valuation of thoughts (Figure 8C). In contrast, when the tonic DA level *τ* is very low, almost all of the individuals suffer from diminished engagement with high-cost activities (Figure 8D); however, this may be based on an overly simplified assumption, and the results depend on the hypothetical population distribution of DA parameters. Maia and Frank (2017) suggested that the aberrant spontaneous DA transients may cause other observable effects, such as neural or behavioral effects responding to neutral stimuli, which correlate with positive symptoms. Using other such behavioral observations may help find that spontaneous DA transients are a cause of the symptoms. Focusing on such behavioral observations that directly reflect the effect of the cause of mental disorders would be one of the core proposals of RDoC.

Note that this simulation is intended only as a simple illustration. In reality, psychosis (including schizophrenia) must be a more complex disorder that may involve several etiological mechanisms. Nevertheless, the present demonstration illustrates the first step to incorporating computational models into the proposed framework to evaluate a psychiatric research strategy.

## DISCUSSION

In this article, we proposed a novel framework for discussing the effectiveness of research strategies in psychiatry. We demonstrated the basic features of the framework considering simple cases. There are many discrepancies between the assumptions of the simple cases and the realistic situations. Before discussing the discrepancies, we discuss the implications derived from the analyses of the model properties.

### Implications

The results of the computer simulations highlight the effectiveness of dissociating a behavioral measure from other behavioral phenotypes that reflect different pathophysiological states. If one uses a diagnostic category whose criteria contain symptoms that can arise from different pathogenetic factors, then the chance of finding the corresponding factor is lowered compared with when one classifies the subjects based on a single symptom separately (as in Cases 1-2 and 3). When two factors have opposite effects on the symptoms (i.e., one factor influences one symptom agonistically, another symptom antagonistically), the statistical power would be weak if both symptoms were used to classify the individuals (Case 4, when the mixture parameter *c* is negative). This may also be true in more realistic situations, as observed in the reinforcement learning model of psychosis (Case 6). The RDoC approach that decomposes the factors and measures into distinct constructs would be promising in this regard.

Meanwhile, the behavioral observations can be contaminated with noise, including errors in the subjective report and individual differences in the reactivity to pathogenetic factors. By using the behavioral observations that share common pathogenetic factors to define the category, the category-based approach can reduce the effect of the noise. Consequently, increasing the number of independent criteria can reduce the impact of such noise and make the detection of the pathogenetic factors easier, given that the errors are mutually independent (as observed in Cases 1-1 and 3).

Therefore, in some cases, the conventional diagnostic category-based approach could be more efficient in detecting a pathogenetic factor than the dimensional (RDoC) approach (as in Case 3). Which approach is better depends on the case. The proposed model provides a promising approach for designing an efficient research strategy to investigate a specific target. By incorporating detailed generative models of psychiatric diseases, the researcher can determine the better research strategy, as we demonstrated in Case 6, which suggests that separating the positive symptoms and negative symptoms is better than treating them as symptoms of a single disorder category (e.g., psychosis).

### Limitations and Possible Extensions

Nevertheless, there are several limitations of the proposed framework. The results and their implications strongly depend on the model assumption. If invalid models are used, then the simulation may recommend a suboptimal or even worse strategy. For example, the selection of the population distribution of model parameters (e.g., DA parameters in Case 6, which we arbitrarily set) might quantitatively change the results. Although the proposed method provides a quantitative prediction, that is, statistical power, keeping the conclusion qualitative would be better. For example, one may draw the conclusion that separating the positive symptom and negative symptom is better for finding the pattern of aberrant DA activity, but one should not trust the specific value of the power to determine the number of subjects to obtain statistically significant results (as an ordinary power analysis does). Seeking an appropriate model of mental disorders itself is a challenging task and within the scope of computational psychiatry.

We have primarily considered the linear Gaussian model as a generative model. This model assumes that the variables take continuous values and obey a Gaussian distribution. Although this assumption makes the theoretical analysis easier, it is an obvious oversimplification. For example, consider a genetic mutation as a pathogenetic factor. The presence or absence of an allele is represented as a categorical variable. The behavioral measure or symptom can also be categorical (e.g., the existence or absence of a specific symptom). The distribution of scores for some symptom ratings can be best explained using an exponential distribution with a cutoff (Melzer, Tom, Brugha, Fryers, & Meltzer, 2002). The use of the link function that maps variables onto the exponential function with a shift parameter may be suitable for such cases. Although the basic properties reported in this study may hold in various other situations, a careful investigation would be needed, depending on the situation.

Another drastic simplification in the present model is the assumption of independence among errors and also among pathogenetic factors. In realistic situations, there may be considerable correlations among them. A second-order correlation can be modeled using a multivariate Gaussian distribution, which is a simple extension of the current model. However, there may be a higher order correlation, for example, in gene expression (Nakahara, Nishimura, Inoue, Hori, & Amari, 2003). Such a correlation structure should be included in the model, depending on the specific situation, particularly for discussing the impact of the relationships between the pathogenetic factors.

### Strategies in Sampling and Analysis

As we have emphasized, the category-based approach and dimensional approach differ in how they sample the subjects. The category-based approach uses diagnostic criteria for selecting the subjects (it samples the “patients”), whereas the dimensional approach is assumed to gather subjects without prior criteria. As shown in Case 2, when the same data (those gathered by the category-based approach) are used, then the dimensional (correlation) approach tends to provide greater statistical power compared to the categorical approach (*t* test). This result suggests that it might be better to use diagnostic criteria when sampling subjects and then perform a dimensional analysis that ignores the diagnostic label. Of course, this also depends on the validity of the model assumption. The critical assumption related to this issue is that the pathogenetic factors and the behavioral observations have a monotonic relation: The greater the measured value of the pathogenetic factor is, the greater the behavioral observation is. Owing to this relation, the severe inclusion criteria for the patient group make their pathogenetic factors easily distinguishable from those of the control group. If there is no such simple monotonic relation, the result can change. Such a violation may occur when the mapping from the pathogenetic factor to behavior has a nonlinear shape, such as the inverted U-shaped function or step function, which has a critical point above which the pathogenetic factor influences the behavior.

On the basis of the proposed framework, one can optimize the inclusion criteria (cutoff point) of specific disorders. As shown in Case 2, the more severe the inclusion criteria of the patient group are, the higher the statistical power is, given our assumption. However, there is a trade-off between the power and difficulty in finding samples. Severe criteria make finding the “patient” individuals difficult. The proposed framework can help determine the criteria considering the trade-off for specific situations. Note that this is the issue of basic research. For practical clinical applications, the optimal criteria would differ. A treatment can be effective even for individuals with modest symptoms. For clinical diagnosis, the optimal boundary should depend on the treatment response rather than be based solely on the statistical power.

### Relation to Computational Psychiatry

We demonstrate how to incorporate the computational model in the proposed framework by using a reinforcement learning model with dopamine dysfunction. Candidate mathematical or computational models can span Marr’s three levels: computational, algorithmic, and implementational (Kurth-Nelson et al., 2016). To discuss how the dysfunction at the cellular or molecular level influences the symptom, models at a detailed biophysical level (e.g., biophysical neural circuit models; Wang & Krystal, 2014) would be suitable, whereas to discuss how the circuit-level dysfunction affects disorders, models at the intermediate level (e.g., connectionist models; Yamashita & Tani, 2012) would be useful. For the computational level or algorithm level, more abstract models, such as the Bayesian cognitive models (e.g., Lee & Wagenmaker, 2014) or reinforcement learning models, are possible candidates. In principle, the proposed framework can incorporate mathematical models at any level, as long as they can represent the rule of translating the pathogenetic factor to behavioral phenotypes. However, the models at a low abstraction level tend to have a large number of variables; thus generating samples for Monte Carlo simulations has considerable computational cost. For such cases, some approximation techniques based on the small number of simulated results would be helpful. For example, the relationship between DA parameters and the fraction of symptoms in Figures 8C and 8D can be approximated using a sigmoid function that is parametrized by these DA parameters. Some model reduction techniques developed in computational neuroscience, such as the mean-field method, would also be helpful (Deco, Jirsa, Robinson, Breakspear, & Friston, 2008; Wang & Krystal, 2014).

Currently the most successful applications of computational approaches to psychiatry may be those based on fitting models to behavior and/or neural activities. For example, model parameters that are fit to the subject’s behavior (e.g., Ahn et al., 2014; Culbreth, Westbrook, Daw, Botvinick, & Barch, 2016; Kunisato et al., 2012; Voon et al., 2015; Yechiam, Busemeyer, Stout, & Bechara, 2005) or the model latent variables that are correlated with blood oxygen level–dependent (BOLD) signals (e.g., Gradin et al., 2011; Murray et al., 2008) have been compared between subjects with a mental disorder and healthy controls. The parameters and variables of computational models are often associated with neuromodulators (Dayan & Huys, 2008; Doya, 2008; Stephan, Iglesias, Heinzle, & Diaconescu, 2015; Yu & Dayan, 2005). If there are indeed such associations, then the estimate of a model parameter or a latent variable can be used as an estimate of a pathogenetic factor. To discuss the effectiveness of such model-based approaches, the model fit procedures can be simulated in our framework: The estimation error of various model fit methods (e.g., maximum likelihood method or hierarchical Bayesian method) can be explicitly examined based on the synthesized dataset generated from a hypothetical “true model” (as in Katahira, 2016).

### Related Work

Recently, Flagel et al. (2016) proposed a novel framework for psychiatric nosology, termed the *Bayesian integrative framework*. This framework includes the generative models that explicitly represent how symptoms, signs, and diagnoses arise from putative causes through a physiological state and “latent constructs,” which correspond to the constructs in RDoC. This framework uses Bayesian inference to infer the posterior probability of latent variables and to evaluate the models from an individual’s data. The procedures of the inference and model selection were demonstrated in Friston (2016).

The Bayesian integrative framework shares a common feature with our framework: Both frameworks use mathematical or computational models to model the generative processes of mental disorders. However, the goals of the two frameworks differ. The Bayesian integrative framework is designed to analyze individual data (including the diagnosis): It infers the latent cause of the disorder of the individuals through Bayesian inference. The Bayesian integrative framework is intended also to be used by clinicians to gather data for improving the model and nosology. The ultimate goals are aiding the diagnosis, prognosis, prevention, and treatment of mental disorders. In contrast, the scope of our framework is basic research strategies in psychiatry rather than a clinical use. In addition, the target of modeling in our framework is the population rather than the individual. Our framework is concerned with the sampling method of subjects, whereas the Bayesian integrative framework does not explicitly deal with the sampling procedure, at least currently. Thus our framework cannot provide useful information about the cause of the disorder of an individual patient, whereas the Bayesian integrative framework would provide such information.

The statistical methods considered in the framework also differ. The Bayesian integrative framework uses the Bayesian method, as its name suggests, whereas our framework is formulated with the classical hypothesis testing in mind, although replacing it with Bayesian hypothesis testing is straightforward. Whereas the Bayesian approach is more flexible, the classical statistical framework is more prevalent in psychiatry studies. The relations between the Bayesian integrative framework and our framework are complementary rather than competing. Our framework would be useful in choosing the research strategy in standard psychiatric studies. As sufficient knowledge is accumulated, more detailed models and precise data become available. Submitting these models and data into the Bayesian integrative framework would help further refine the model and predict treatment outcomes for individual patients.

## CONCLUSION

Psychiatry targets extremely complex processes and systems, that is, mental processes and the brain. Many factors are involved in these processes and systems. Accordingly, there should be various research strategies in psychiatry, as well as in neuroscience and psychology. A framework for evaluating the research strategies is required. Discussion at the verbal description level is limited because the target system is very complex and may not be fully described verbally. Thus computational and mathematical models could play important roles. Although there is plenty of room for modifications, the present study is a first step toward such theoretical evaluations. Our study also provides an avenue via which computational approaches can contribute to psychiatric research.

## AUTHOR CONTRIBUTIONS

K.K. and Y.Y. conceived and designed research; K.K. conducted simulations and analysis; K.K. and Y.Y. wrote the paper.

## FUNDING INFORMATION

This work was supported by JSPS KAKENHI grants JP15K12140, JP25330301, JP17H05946, and JP17H06039 and by JST CREST grant JPMJCR16E2.

## ACKNOWLEDGMENTS

The authors thank Tsukasa Okimura, Yoshihiko Kunisato, and Asako Toyama for their helpful discussion and constructive comments on this study.

## Note

^1^ The Monte Carlo simulation to obtain the power was performed 100,000 times for each condition. We confirmed that the confidence intervals of the power estimate were less than 0.01 (the confidence intervals are drawn on the figure, although they are almost invisible). Thus the estimated powers are highly reliable.
